# Mapping and classifying molecules from a high-throughput structural database

**DOI:** 10.1186/s13321-017-0192-4

**Published:** 2017-02-02

**Authors:** Sandip De, Felix Musil, Teresa Ingram, Carsten Baldauf, Michele Ceriotti

**Affiliations:** 1 National Center for Computational Design and Discovery of Novel Materials (MARVEL), Lausanne, Switzerland; 2 Laboratory of Computational Science and Modelling, Institute of Materials, Ecole Polytechnique Fédérale de Lausanne, Lausanne, Switzerland; 3 Theory Department of the Fritz Haber Institute, Faradayweg 4-6, 14195 Berlin-Dahlem, Germany

## Abstract

High-throughput computational materials design promises to greatly accelerate the process of discovering new materials and compounds, and of optimizing their properties. The large databases of structures and properties that result from computational searches, as well as the agglomeration of data of heterogeneous provenance leads to considerable challenges when it comes to navigating the database, representing its structure at a glance, understanding structure–property relations, eliminating duplicates and identifying inconsistencies. Here we present a case study, based on a data set of conformers of amino acids and dipeptides, of how machine-learning techniques can help addressing these issues. We will exploit a recently-developed strategy to define a metric between structures, and use it as the basis of both clustering and dimensionality reduction techniques—showing how these can help reveal structure–property relations, identify outliers and inconsistent structures, and rationalise how perturbations (e.g. binding of ions to the molecule) affect the stability of different conformers.

**Electronic supplementary material:**

The online version of this article (doi:10.1186/s13321-017-0192-4) contains supplementary material, which is available to authorized users.

Computational materials design promises to greatly accelerate the discovery of materials and molecules with novel, optimized or custom-tailored properties. With this goal in mind, several community efforts have emerged over the past few years [1–8] that aim at generating, and/or storing large amounts of simulation data in publicly available databases [9–15]. The development of these repositories of structural data, and of associated materials properties (e.g. formation energy, band gap, polarizability, ...) poses considerable challenges, from the points of view of guaranteeing consistency, accuracy and reliability of the stored information, as well as that of extracting intuitive insight onto the behavior of a given class of materials and of data-mining in search of compounds that exhibit the desired properties or that are somehow interesting or unexpected.

In order to automate these tasks—which is necessary to unlock the full potential of computational materials databases that can easily contain millions of distinct structures—a number of different machine-learning algorithms have been developed, or adapted to the specific requirements of this field [16–25]. A fundamental ingredient in all of these approaches is a concise mathematical representation of a molecular or crystalline structure, that can take the form of fingerprints (low-dimensional representation of the structure of the atoms) or more abstract measures of the (dis)similarity between elements in the database, such as distance or kernel functions.

In the present manuscript we will present a demonstration of how a very general approach to quantify structural dissimilarity [26] can be combined with non-linear dimensionality reduction and clustering techniques to address the challenges of navigating a database of molecular conformers, checking its internal consistency and rationalising structure–property relations. Even though we will focus in particular on a energy/structure data set of amino acid and dipeptide conformers obtained by an ab initio structure search [15, 27], many of the observations we will infer are general, and provide insight on the application of machine-learning techniques to the analysis of molecular and materials databases generated by high-throughput computations.

## A toolbox for database analysis

**Fig. 1:**
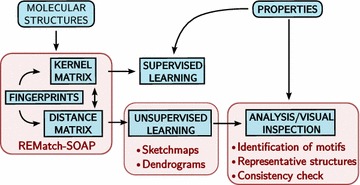
A flowchart summarizing the different ingredients that enter a semi-automated workflow for the representation and analysis of a database of atomistic structures. We highlight with a *blue background* the general components, and with a *red background* the methods and applications we explore in the present work

Automatic analysis of atomistic structures obtained from large databases of materials and molecules requires a combination of different techniques (Fig. 1). A representation of structures in terms of “fingerprints”, distances or kernels serves as the input of unsupervised-learning techniques (clustering, dimensionality reduction, …) that greatly simplify the verification of the database for internal consistency, and the identification of organising principles and structure/property relations. Although we will not discuss this aspect explicitly here, molecular representations can also be used to directly predict properties using supervised learning techniques such as kernel-ridge regression or neural networks. In this section we will describe a specific combination of descriptors and unsupervised-learning algorithms, but we will also briefly summarize some of the alternative approaches that could be used to substitute different components of our tool chain.

### Fingerprints and structural similarity

The most crucial and basic element in any structural analysis algorithm is to introduce a metric to measure (dis)similarity between two atomic configurations. Many options are available, with different levels of complexity and generality, starting from the commonly used root mean square (RMS) distance. In order to deal with symmetry operations or condensed phase structures, several “fingerprint” frameworks have been developed [8, 28–40], that assign a unique vector of order parameters to each molecular or crystalline configuration: a metric can then be easily built by taking some norm of the difference between fingerprint vectors. Any of these distances could be taken as the basis of the classification and mapping algorithms that we will describe in what follows.

In this paper we will use instead a very flexible framework (REMatch-SOAP) that is based on the definition of an environment similarity matrix $C_{ij}(A,B)$, which contains the complete information on the pair-wise similarity of the environment of each of the atoms within the molecules *A* and *B*. In our framework, the similarity between two local environments ${\mathscr {X}}^A_{i}$ and ${\mathscr {X}}^B_{j}$ is computed using the SOAP kernel [29]

1 $$C_{ij}(A,B)=k\left( {\mathscr {X}}^A_{i},{\mathscr {X}}^B_{j}\right) .$$

The REMatch kernel is then defined as the following weighted combination of the elements of ${\mathbf {C}}(A,B)$

2 $$\begin{aligned} {\hat{K}}^{\gamma }(A,B)&= {\text {Tr}}{\mathbf {P}}^\gamma {\mathbf {C}}(A,B), \\ {\mathbf {P}}^\gamma&= \mathop {\mathrm{argmin}}\limits _{{\mathbf {P}}\in {\mathscr {U}}(N,N)} \sum _{ij} P_{ij} \left( 1-C_{ij}+\gamma \ln P_{ij}\right) , \\ C_{ij}(A,B)&= k\left( {\mathscr {X}}^A_{i},{\mathscr {X}}^B_{j}\right) , \end{aligned}$$

where the optimal combination is obtained by searching over the doubly stochastic matrices ${\mathscr {U}}(N,N)$ the one that minimizes the discrepancy between matching pairs of environments subject to a regularization based on the matrix information-entropy $E({\mathbf {P}})=-\sum _{ij}P_{ij}\ln P_{ij}$ [41]. Once a kernel between two configurations has been defined, it is possible to introduce a kernel distance

3 $$D(A,B)=\sqrt{{\hat{K}}^{\gamma }(A,A)+{\hat{K}}^{\gamma }(B,B)-2{\hat{K}}^{\gamma }(A,B)},$$

that we will use as the metric for representing and clustering structures from a database.

As discussed in Ref. [26], the choice of a SOAP kernel as the definition of an environment similarity provides at the same time great generality—it can be seamlessly applied to both molecules and solids—and elbowroom for fine-tuning—ranging from setting an appropriate cutoff distance to circumscribe an environment to the introduction of an alchemical similarity kernel that translates the notion that different chemical species can behave similarly with respect to the properties of interest.

### Mapping the structural landscape of a database

The dissimilarity between the *N* atomic configurations in a database contains a large amount of information on the structural relations between the database items. However, this information is not readily interpretable, as it is encoded as a $N^2$ matrix of numbers. Several methods are available to process dissimilarity information into a form that can be understood more intuitively. A first approach involves building a low-dimensional “map”, where each point corresponds to one of the structures in the database and where the (Euclidean) distances between points represents the information on the pairwise dissimilarity matrix.

Several methods have been proposed over the years to solve this dimensionality reduction problem, starting from principal component analysis [42] and the equivalent linear multi-dimensional scaling [43], and proceeding to non-linear generalizations of the idea, such as ISOMAP [44], diffusion maps [45], kernel PCA [46]. In this manuscript, we will use sketchmap [22–24], a method in which one iteratively optimizes the objective function

4 $$S^2 = \sum _{ij} \left[ F\left[ D(X_i,X_j)\right] - f\left[ d(x_i,x_j)\right] \right] ^2,$$

that measures the mismatch of the dissimilarity between atomic configurations $D(X_i,X_j)$ with the dissimilarity (typically just the Euclidean distance) between the corresponding low-dimensional projections $\left\{ x_i\right\}$. The procedure is very similar to multi-dimensional scaling, except for the appearance of the transformations *F* and *f*, which are non-linear sigmoid functions of the form:

5 $$F(r) = 1 - \left( 1 + (2^{a/b} - 1)(r/\sigma )^a \right) ^{-b/a}.$$

The non-linear transformation focuses the optimization of Eq. (4) on the most significant distances (typically those of the order of $\sigma$), and disregards local distortions (e.g. induced by thermal fluctuations or by incomplete convergence of a geometry optimization) and the relation between completely disconnected portions of configuration landscape. The maps that we report in this work will be labeled synthetically using the notation $\sigma$–A_B–a_b, where *A* and *B* denote the exponents used for the high-dimensional function *F*, *a* and *b* denote the exponents for the low-dimensional function *f*, and $\sigma$ the threshold for the switching function. The choice of these parameters of the sigmoid functions are discussed in detail elsewhere [24]. In practice *A*, *B*, *a* and *b* have relatively small effect on the projection, and can be optimized and kept fixed for systems belonging to the same family. Since the structures we consider here are minimum-energy configurations, and there are no thermal fluctuations that should be filtered out, we set $A=a=1$ (so that at short range the algorithm will still try to represent distances faithfully) and set the long-range exponents to $B=b=4$. The parameter $\sigma$ is the one to which sketchmap is most sensitive, and needs to be tuned for each system separately. To automate the process of building sketchmaps of large amount of subsets of the database, we have used a simple heuristic procedure for determining the value of $\sigma$ automatically. Following the prescriptions in Ref. [24], we first compute the histogram of distances in the dissimilarity matrix of each molecular set, and detect the dissimilarity value ($D_{max}$) corresponding to the peak value of the histogram. We then set the value of $\sigma$ to $0.8D_{max}$.

### Hierarchical clustering representation

As we will demonstrate below, sketchmap provides a remarkably informative two-dimensional representation of structures in a data set, making it possible to identify groups of similar configurations, outliers, as well as to investigate structure–property relations. An alternative approach to navigate a set of structures based on the dissimilarity matrix is to use clustering algorithms, that identify groups of objects having similar properties to hint at the presence of recurring motifs underlying the behavior of the system.

A considerable number of clustering algorithms have been developed over the last few decades [17, 47, 48], including connectivity models [49] (i.e. hierarchical clustering), centroid models [50–52] (i.e. k-means algorithm) and density based models [16, 53, 54].

Clustering models based on connectivity information such as hierarchical (or agglomerative) clustering [49] are particularly suited for this purpose and we will focus only on this type of clustering in this paper. Starting from each configurations as its own cluster, the hierarchical clustering algorithm iteratively aggregates clusters together based on some assessment of their dissimilarity. Dissimilarity between two individual structures can be obviously measured by their distance *D*(*A*, *B*). The distance between two *clusters*, however, can be defined in many different ways. In our study, we will use in particular the RMS dissimilarity between the pair of members of the two clusters. The linkage distance $\Delta$ between two clusters ${\mathbb {X}}=\left\{ X_i\right\}$ and ${\mathbb {Y}}=\left\{ Y_i\right\}$ is then defined as:

6 $$\Delta ({\mathbb {X}},{\mathbb {Y}})= \sqrt{ \frac{1}{N_{\mathbb {X}} N_{\mathbb {Y}}}\sum _{X \in {\mathbb {X}} , Y \in {\mathbb {Y}}}{D^2(X,Y)} },$$

where $N_{\mathbb {X}}$ and $N_{\mathbb {Y}}$ are the total number of configurations within each cluster. *D*(*X*, *Y*) is the dissimilarity between the two configurations, that in our case was computed based on the REMatch-SOAP kernel. The complexity of this type of clustering in terms of the number of structures *N* is relatively cheap (${\mathscr {O}}(N^2log(N))$) compared with dimensionality reduction algorithms like sketchmap (${\mathscr {O}}(N^3)$). Both procedures can be greatly accelerated through out of sample embedding. A subset of the configurations is selected (e.g. by farthest point sampling, with the possibility of weighting based on density information [24]) for either dimensionality reduction or hierarchical clustering and then is used as a reference for the projection/clustering of the other structures.

The results of a hierarchical clustering procedure can be represented in a “dendrogram” plot, that conveys visually the sequence of agglomerative clustering operations and the linkage distance at each step. The lowest level of the dendrogram is composed of single-structure clusters, so that the *x* axis corresponds to individual configurations sorted according to the clustering procedure. Each merge operation is represented by a line joining the two underlying clusters, with the *y* position of the line representing the linkage distance for that pair, as defined by Eq. (6). In this kind of representation, at the bottom of the dendrogram, each structure can be thought of as an individual cluster containing only one item. Clusters are then merged iteratively, selecting at each step the pair of clusters that are closest to each other. This operation is repeated until all the clusters collapse into one single group that encompasses all the structures in the database, thus completing the dendrogram. To avoid overcrowding the bottom of the plot, one can hide the part that corresponds to very small linkage distances, while still graphically visualising the size of the clusters by drawing bars that encompass the associated structures. Since the “leaves” of this dendrogram correspond to individual configurations, it is possible to complement the dendrogram with color-coded bar plots that represent the value of different properties of each structure, thereby giving a clear picture of the relation between structural clustering and the different properties.

In order to understand the basic motifs of a particular cluster ${\mathbb {X}}$, it is very useful to select one of its structures that is as representative as possible of the entire subset. In case where stability estimates are available, such structure may be the lowest-energy structure in the cluster. For a definition that is based purely on conformational or configurational information, the most representative structure $RS\left( {\mathbb {X}}\right)$ could be defined as the item having the minimum mean square dissimilarity with respect to all other members of ${\mathbb {X}}$, i.e.

7 $$\begin{aligned} RS\left( {\mathbb {X}}\right) = \mathop {{{\mathrm{argmin}}}}\limits _{X_1\in {\mathbb {X}}}\left[ \frac{1}{N_{\mathbb {X}}}\sum _{X_2\in {\mathbb {X}}}D^2(X_1,X_2) \right] . \end{aligned}$$

Representative structures can be defined at each level of the hierarchy, and can therefore be very useful in navigating the database, and understanding what are its most crucial structural features. The spread of the cluster around $RS\left( {\mathbb {X}}\right)$,

8 $$\begin{aligned} \sigma _D\left( {\mathbb {X}}\right) = \sqrt{\frac{1}{N_{\mathbb {X}}}\sum _{X\in {\mathbb {X}}}D^2(X,RS({\mathbb {X}})) }, \end{aligned}$$

can be used to quantify the range of structural landscape that is covered by the cluster.

Another important aspect of database analysis is outlier detection [55–60]. An outlier configuration is defined as a configuration which is different from most of the configurations in the database. Outlier configurations are very important as they are likely to have unique structural motif in the whole database and are thus interesting for structure prediction applications. They also could represent chemical changes or indicate inconsistent configurations which are likely to be “errors” in the database.

In the following sections, we will present examples of how these different analyses can be applied to different subsets of structures taken from a database of amino acid and dipeptide conformers.

## Analysis of a database

This work is based on a first-principles derived structure/energy data set with conformers of twenty proteinogenic amino acids and dipeptides, as well as their interactions with a series of divalent cations [27] (Ca^2+^, Ba^2+^, Sr^2+^, Cd^2+^, Pb^2+^, Hg^2+^). The potential-energy surfaces (PES) of 280 systems were explored in a wide relative energy range of up to 4 eV (390 kJ/mol), summing up to an overall of 45,892 stationary points on the respective potential-energy surfaces [15]. The underlying energetics were calculated by applying density-functional theory (DFT) in the generalized gradient approximation corrected for long-range van der Waals interactions [61–63] (PBE + vdW). A number of theory-theory and theory-experiment comparisons have shown the applicability of the method to amino acid and peptide systems [15, 64–69]. The generation of this dataset involved significant manual intervention, and one would expect it to be an easy starting point for studying materials and molecules across chemical space [70]. Nevertheless, we will demonstrate that, even for such heavily curated data, automated techniques are needed to extract unbiased and hypothesis-free trends and to check for internal consistency.

**Fig. 2:**
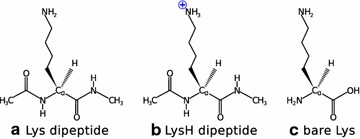
The lysine building block was studied in three forms: **a** uncharged dipeptide, **b** protonated dipeptide, and **c** uncapped and uncharged amino acid

In this study we focus on the amino acid lysine (in short Lys) and investigate basic structural motifs of three forms, see Fig. 2. Furthermore, the machine learning techniques introduced in this work are used to detect the impact of perturbations (here Ca^2+^ cations) on the structural properties of the unperturbed systems. Finally, we demonstrate how the approach can also be applied to discover inconsistencies and outliers in the database. Hierarchical classifications and sketchmap projections for all the proteinogenic amino acids in the database are given in the Supporting Information.

### Finding the dominant features of a structual landscape

#### Lysine dipeptide

**Fig. 3:**
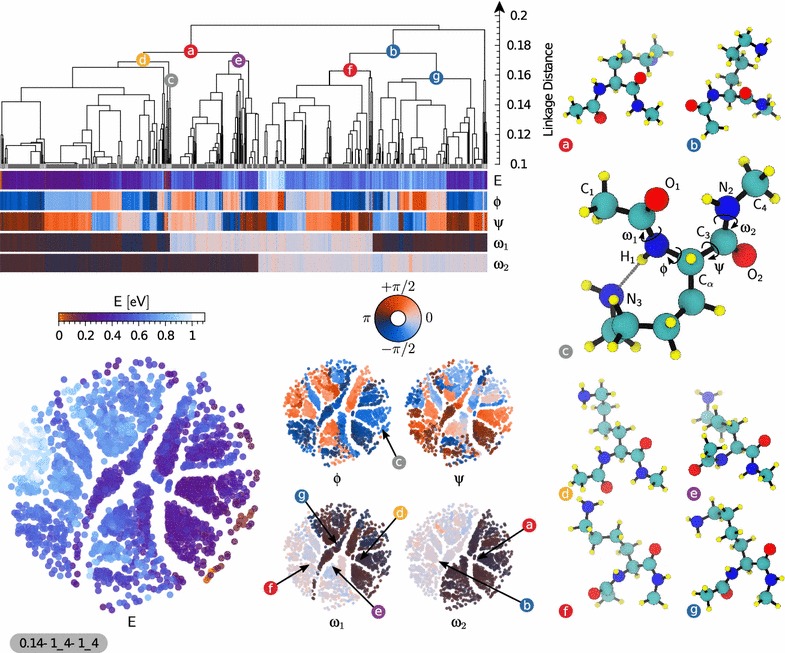
Representation of the similarity matrix corresponding to the lysine dipeptide dataset using the agglomerative clustering algorithm (*top*) and the sketchmap algorithm (*bottom*, projection parameters shown following the scheme $\sigma$–*A*_*B*–*a*_*b*). A few representative structures (see Eq. (7)) of interesting clusters are shown (*right*) and their corresponding position on the sketchmaps and dendrogram representation is *highlighted*. The five sketchmaps are colored according to the conformational energy and the backbone dihedral angles ϕ, ψ, ${{\upomega }}_1$ and ${{\upomega }}_2$. The dendrogram shows the clustering hierarchy of the structures of the dataset. Each structure is vertically aligned with its properties shown using *color bars* below the dendrogram. The dendrogram is cut at a linkage distance of 0.1 since structural properties are very similar below this threshold, and the clusters that are merged at this level are shown as *thick gray bars* separated by *light-gray lines*. Clusters composed of only one structure are drawn as a *black line* reaching the bottom of the dendrogram. The main structural motifs of this set of structures are governed by the peptide bond dihedral angles ${{\upomega }}_1$ and ${{\upomega }}_2$. The two main clusters **a**, **b** are showing a global correlation with the angle ${{\upomega }}_2$ while the angle ${{\upomega }}_1$ splits them into two well correlated sub-clusters (**d**)–(**g**) respectively. The cluster **c** is highlighted as an example containing ‘outlier’ structures of low conformational energy

We take as our first example a subset of the database containing 2080 conformers of lysine dipeptide. As discussed in the previous section, we start by constructing the (dis)similarity matrix using the SOAP-REMatch kernel. In Fig. 3 the dendrogram plot as well as sketchmaps have been shown along with five properties, energy and four dihedral angles, using the same color scales in both the sketchmap and dendrogram representations. In the sketchmap each circular ‘disk’ represents a conformer. Whereas in the case of the dendrogram plot, structures are represented by vertical lines at the bottom of the plot. The strong correlation between energy and conformational parameters on one side, and clustering and position on the map on the other, testifies how the the REMatch-SOAP kernel induces a meaningful classification of the structures in this dataset.

While both clustering and sketchmap show clearly that the dataset is composed of groups of structurally-related conformers, the agnostic nature of the underlying metric does not disclose immediately the structural features that most transparently differentiate between different clusters. Comparing the representative structures from the main clusters allowed us to quickly identify candidate structural motifs that could be used to rationalize the layout of the conformational landscape. By color-coding the dendrogram and the sketchmaps according to these indicators one can readily highlight the key correlations. When considering existing literature on the stability of oligopeptides, the two structural parameters that are most often considered as the key coordinates to navigate the conformational landscape are the Ramachandran dihedral angles ϕ and ψ, that determine the structure of the backbone around the side chain bearing ${\hbox {C}}{\upalpha }$ atom [71] under the assumption of peptide bonds being solely in *trans* conformation. While fine-grained clusters are homogeneous with respect to the ϕ and ψ angles, it is clear that for the present systems the clear-cut branching at the top of the dendrogram is determined by some other order parameter. An analysis of the representative structures for the two main clusters (a) and (b) shows that the two molecules differ by the isomerization of the N-terminal peptide bond. Further splitting of these two clusters, i.e. (a) into clusters (d) and (e), and (b) into (f) and (g), depends on the isomerization of the C-terminal peptide bond. We can confirm this attribution of the main features of the dataset by color-coding the map and the dendrogram following the dihedral angles ${{\upomega }}_1$ and ${{\upomega }}_2$. The four main clusters are largely homogeneous with respect to peptide bond isomerization, and are then further subdivided based on ϕ and ψ. This observation deserves some further comment. Peptide bonds in naturally-occurring proteins are believed to almost exclusively exist in *trans* conformation with the exception of prolyl peptide bonds where a smaller energy difference to *trans* increases the chance for *cis* conformers [72, 73]. This view is supported by the analysis of protein structures deposited in the protein databases where *cis* conformations are found for about 5% of the prolyl peptide bonds, but less than 0.1% for the others [74]. X-ray crystallographic structure represent however merely frozen snapshots of structural dynamics. The ab initio structure search protocol, instead, does consider the peptide bond torsions as variable and intentionally allowed simulations to overcome the isomerization barrier. Consequently, the dataset contains representatives of all four combinations of *cis* and *trans* conformers. Since these transitions are strongly bimodal, and reflect in significant changes of the favorable side chain conformations, they constitute the most significant feature to classify the conformers. As expected, the most stable conformers are largely in a *trans*–*trans* conformation. However, the large parts of conformational space that is occupied by conformers with 1 or 2 *cis* peptide bonds suggests that *cis* isomers might play a role in the dynamics of peptides and proteins. Consequently, an analysis only focused on the Ramachandran dihedrals, ϕ and ψ, would have missed one of the main features of the structural landscape that is critical to characterize the relation between structure and energetics. One could then proceed further with the analysis, focusing for instance on small clusters containing low-energy structures such as that represented by the conformer (c). All the structure in this group are *trans*–*trans* isomers, that in addition have ${\upphi }{}\approx -90$° and ${\uppsi }{}\approx 90$°, allowing for the formation of a H-bond between the side chain $\hbox {N}_{3}$ and $\hbox {H}_{1}$, and a favorable arrangement of the $\hbox {N}_{2}$ donating a H-bond to the carbonyl $\hbox {O}_{1}$ as shown in Fig. 3. Having access to the combined information on energetics, and on the grouping of structures with similar geometry makes it easier to rationalize the energy ordering of the structures, without having to separately juxtapose all the low-lying conformers but focusing on a few representative configurations.

#### Protonated lysine dipeptide

**Fig. 4:**
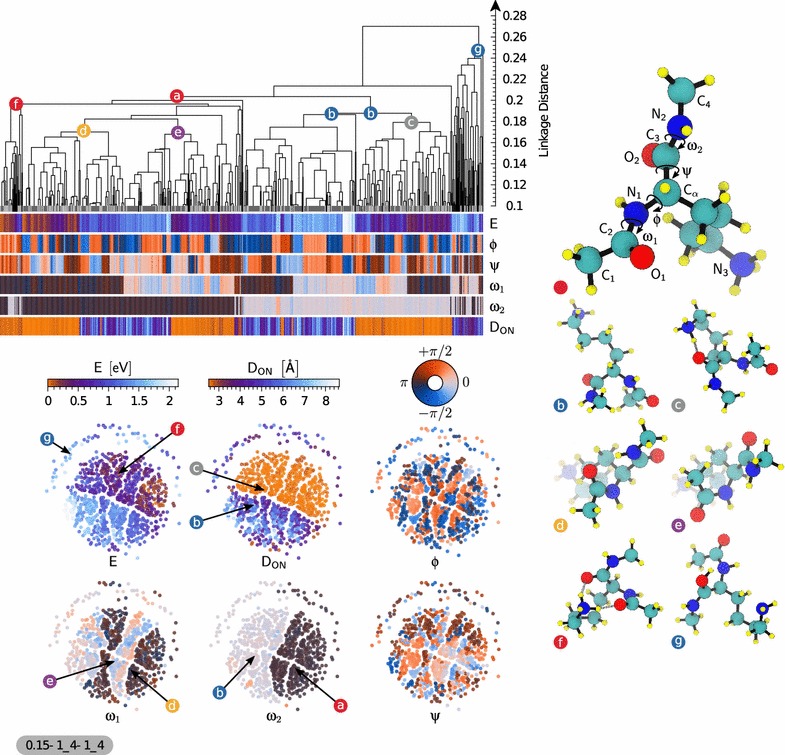
Representation of the similarity matrix corresponding to the protonated lysine dipeptide dataset using the agglomerative clustering algorithm (*top*) and the sketchmap algorithm (*bottom*, projection parameters shown following the scheme $\sigma$–*A*_*B*–*a*_*b*). A few representative structures (see Eq. 7) of interesting clusters are shown (*right*) and their corresponding position on the sketchmaps and dendrogram representation is *highlighted*. The six sketchmaps are colored according to the conformational energy, the minimal distance between $\hbox {O}_{1}$ or $\hbox {O}_{2}$ with $\hbox {N}_{3}$ called $\text {D}_{\text {ON}}$, and the backbone dihedral angles ϕ, ψ, ${{\upomega }}_1$ and ${{\upomega }}_2$. The dendrogram shows the clustering hierarchy of the structures of the dataset. Each structure is vertically aligned with its properties shown using *color bars* below the dendrogram. The dendrogram is cut at a linkage distance of 0.1 since structural properties are very similar below this threshold, and the clusters that are merged at this level are shown as *thick gray bars* separated by *light-gray lines*. Clusters composed of only one structure are drawn as a *black line* reaching the bottom of the dendrogram. The main structural motifs of this set of structures are governed by the dihedral angles ${{\upomega }}_1$ and ${{\upomega }}_2$ and the distance $\text {D}_{\text {ON}}$. The two main clusters **a**, **b** are showing a global correlation with the angle ${{\upomega }}_2$ while the angle ${{\upomega }}_1$ splits them into well correlated sub-clusters (e.g. sub-clusters **d**, **e**). The other important sub-clustering parameter is the distance $\text {D}_{\text {ON}}$, e.g. sub-clusters (**c**) and (**b**), which also correlates well with the separation between low and high conformational energy shown on the sketchmaps. Two sub-clusters are particular: **g** is a clear ‘outlier’ due to a chemical change and **f** features a H-bonding pattern with the side chain NH$_3^+$ pointing to both carboxy groups that sets this cluster apart from all others

As the second example we considered a dataset containing 897 conformers of gas phase protonated lysine dipeptide. We follow the same steps as described in the previous example in order to find the most basic structural motifs of this system. Figure 4 shows the dendrogram, the sketchmap and a few color coded properties of this system to demonstrate their correlation with the classification. The most prominent feature for this molecule, which is evident in both the dendrogram and the sketch maps, is the presence of a group of outliers, that are clearly separated from the bulk of the conformers. Inspection of the cluster centroid (g) clarifies the structural basis of this separation. Whereas in most of the structures the excess charge lies on the lysine side chain as a NH$_3^+$ group, conformers in this cluster experienced a proton transfer event, with the excess proton attached to one of the carbonyl oxygen $\hbox {O}_{1}$, stabilized by H-bonding to $\hbox {N}_{2}$. This is a result of the database generation where ab initio replica-exchange molecular dynamics including high T trajectories where used for structure sampling during which protons can eventually transfer.

Moving on to the main cluster of structures, we can see that similar to our previous example of the neutral dipeptide and again due to the unbiased sampling protocol and the high energy range the peptide bond angles are again more important than Ramachandran’s dihedrals. Conformers (a) and (b) are the representative structure for groups having *cis* and *trans* ${{\upomega }}_2$ peptide bonds respectively. Group (a) is further split based on the *cis/trans* state of ${{\upomega }}_1$ into the clusters represented by structures (d) and (e).

The presence of a charged side-chain leads to stronger H-bonds. As a consequence, peptide-bond isomerism plays a less crucial role in determining structural clustering than for the neutral dipeptide. An example of the importance of H-bonds is given for instance by the subcluster represented by conformer (f), in which the bent side chain leads to the formation of two H-bonds between NH$_3^+$ group and the carbonyl oxygens. H-bonds also dominate the partitioning of cluster (b), that is split into two groups—one of which is still best represented by the same conformer, and one that is epitomised by (c). Once again, inspection of these structural representatives reveals the organising principle behind the classification: (c)-like structures have an extended side chain, and are dominated by interactions among the peptide bond moieties, whereas (b)-like structures have a well-formed H-bond between the side chain and one of the two backbone O atoms. This structural pattern can be emphasized by color-coding conformers based on the parameter $\text {D}_{\text {ON}}=\min \left[ d(\hbox {O}_{1},\hbox {N}_{3}); d(\hbox {O}_{2},\hbox {N}_{3})\right]$: A small O-N distance indicates bending of the side chain and the formation of a H-bond between O and N. As it is clear from the sketchmap representation, there is a very strong correlation between the bending of the charged side chain and the energy of a conformer. All of the structures within 0.5 eV of the ground state feature this sidechain to backbone H-bonds.

It is worth noting that the importance of intramolecular H-bonds is a consequence of the gas-phase environment in which the structure search was performed. In a polar solvent like water, where intramolecular H-bonds that introduce strain compete with H-bonds with the surrounding water molecules, that do not require a bending of the side-chain, the energy balance might be different or less clear-cut. The analysis techniques we introduce in this work would be ideally suited to rationalize the changes in the (free) energetics of biological molecules when moving from the gas phase to (micro)solvated environments or to organic/inorganic interfaces.

#### Uncapped lysine

**Fig. 5:**
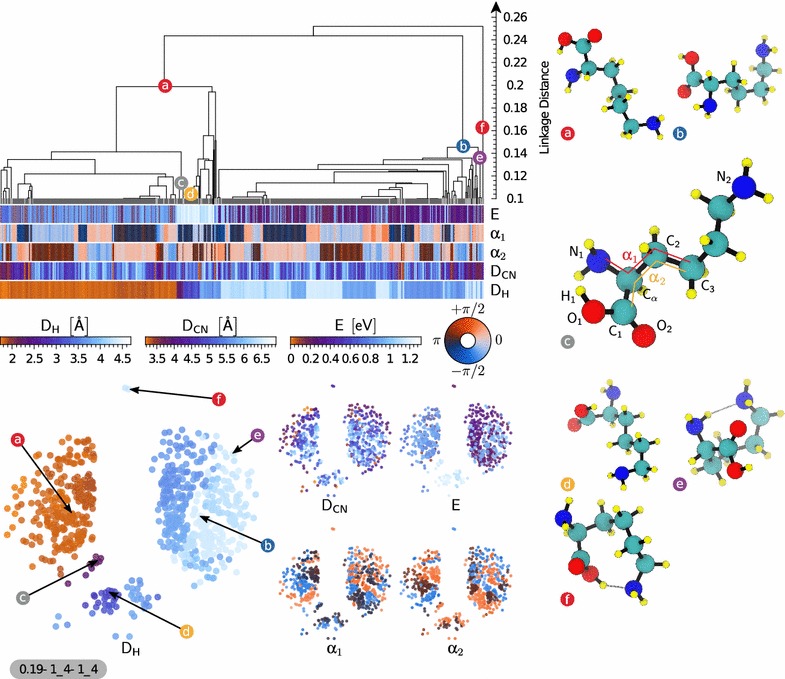
Representation of the similarity matrix corresponding to the lysine uncapped dataset using the agglomerative clustering algorithm (*top*) and the sketchmap algorithm (*bottom*, projection parameters shown following the scheme $\sigma$–*A*_*B*–*a*_*b*). A few representative structures (see Eq. 7) of interesting clusters are shown (*right*) and their corresponding position on the sketchmaps and dendrogram representation is *highlighted*. The five sketchmaps are colored according to the conformational energy, the distance between $\hbox {N}_{1}$ and the hydrogen in the carboxilic group $\hbox {H}_{1}$ (labelled $\text {D}_{\text {H}}$), the distance between $\hbox {N}_{2}$ and $\hbox {C}_{\alpha }$ (labelled $\text {D}_{\text {CN}}$), and the dihedral angles ${\upalpha }_{1}$ and ${\upalpha }_{2}$ which are respectively computed with the following atoms ($\hbox {N}_{1},\hbox {C}_{\alpha },\hbox {C}_{2},\hbox {C}_{3}$) and ($\hbox {C}_{1},\hbox {C}_{\alpha },\hbox {C}_{2},\hbox {C}_{3}$). The dendrogram shows the clustering hierarchy of the structures of the dataset. Each structure is vertically aligned with its properties shown using *color bars* below the dendrogram. The dendrogram is cut at a linkage distance of 0.1 since structural properties are very similar below this threshold, and the clusters that are merged at this level are shown as *thick gray bars* separated by *light-gray lines*. Clusters composed of only one structure are drawn as a *black line* reaching the bottom of the dendrogram. The main structural motifs of the database are governed by the distance $\text {D}_{\text {H}}$. The two main clusters **a**, **b** are agglomerated according to the orientation of $\hbox {H}_{1}$ and the oxygen atom it is bonded to with respect to $\hbox {N}_{1}$ which is well described by the distance $\text {D}_{\text {H}}$. The sub-cluster **e** is composed of ‘outlier’ structures showing an H-bond between $\hbox {N}_{2}$ and an hydrogen of $\hbox {N}_{1}$ resulting in a folded side chain structural motif. Finally, the outlier cluster **f** contains a H-bond between the carboxy H and the side-chain $\text {NH}_{2}$, that can be seen as a precursor to the zwitterionic form

Our third example is a dataset containing 733 conformers of the un-capped lysine molecule in the gas phase. We follow the same steps as described in the previous examples to construct the dendrogram shown in Fig. 5. The map has a simple structure, with few well-separated groups. Being a smaller molecule with fewer degrees of freedom, the Ramachandran angles are not defined. The dihedral angles in the vicinity of the $\hbox {C}_{\alpha }$ atom display local structural correlation but once again they are not the main organizing factor that can rationalize the clustering. By juxtaposing representative conformers from the main clusters we could identify a better order parameter, that correlates strongly with H-bond patterns within the molecule. Namely, the distance ($\text {D}_{\text {H}}$) between the H atom in the OH group of the carboxyl function and the N atom in the backbone (N_1_) discriminates well between structures based on H-bonding patterns [70] of *type I* between $\hbox {N}_{1}\hbox {H}_{}\rightarrow \hbox {O}_{2}$ (e.g. conformer (b)) and of *type II* with a H-bond $\hbox {O}_{1}\hbox {H}_{}\rightarrow \hbox {N}_{1}$ (e.g. conformer (a)). It can be seen from both the dendrogram and the sketchmaps that one could identify several subgroups based on particular values of $\text {D}_{\text {H}}$, representing specific orientations. Conformers (c) and (d) represent small groups of conformers having specific relative orientation between the OH and $\text {NH}_{2}$ groups. Conformer (e) is representative of a small outlier group with a well-defined bend of the side chain, leading to the formation of a further H-bond between the $\hbox {N}_{1}$ atom in the amino acid moiety and $\hbox {N}_{2}$, in the side chain. The lysine side chain is very flexible, and the distance between N and $\hbox {C}_{\alpha }$ only plays a role in defining the fine-grained structure of the dataset, but is minimally correlated with the most prominent features.

**Fig. 6:**
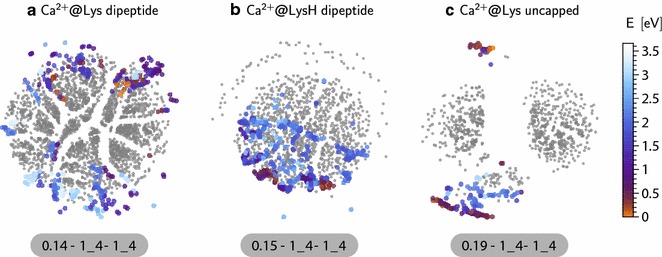
The out-of-sample embedding of conformers with Ca^2+^ ion on the sketchmap of their pure counterpart, for the three systems we discussed in above: lysine dipeptide (**a**), protonated lysine dipeptide (**b**) and molecular lysine (**c**) systems. The projected conformers are colored with their energy where as the sketchmap on which they are projected are kept all in *grey color*. The location of the projected conformers allows us to understand how the conformational space of the pure conformers are affected due to presence of the Ca^2+^ ion

While it appears that even in this case we could identify the basic structural motifs that characterize the conformational landscape of this system, the correlation with energy is very poor. There are several instances, in both the dendrogram and the sketchmap, where two conformers that are detected as structurally very similar display very different stability. Understanding whether this inconsistency signals a problem with our analysis brings us to the topic of outlier detection and consistency checks, that we will discuss in details in the “Identifying outliers and checking for consistency” section.

### Understanding the impact of perturbations on conformational space

Having elucidated the essential structural motifs that underlie the organization of a set of molecular conformers, one could also wonder how changes in the thermodynamic conditions, or other external perturbations such as solvation, the addition or subtraction of an electron [75] or that of an atom [76–78], modify the conformations of the molecule and their stability. In addition to bare oligopeptides, the database [15, 27] that we are using as an example contains sets of locally-stable conformers in the presence of cations of six different species, namely Ca^2+^, Ba^2+^, Sr^2+^, Cd^2+^, Pb^2+^ and Hg^2+^. We consider the case of Ca^2+^ to describe how one can characterize its impact on the conformational space of the three molecular systems that we have discussed in our previous examples. We start by calculating the dissimilarity of all the conformers containing cations with their pure counterpart. In order to make the comparison on the same footings, we did not include the location of the cation in defining the SOAP kernels, so that the presence of Ca^2+^ only enters by distorting molecular geometries and/or altering their relative stability. Using this information, we then projected the cation-containing dataset on the top of the sketchmap of structures for the bare molecule. This is done using sketchmap out-of-sample embedding, and we refer our reader to see the relevant literature [22–24] for more details about the method. In Fig. 6 we show the resulting projection, colored according to the stability of the conformers, on top of the sketchmap of the pure molecule shown in grey color as a reference. A close proximity of projected conformers with a pure conformer signifies their structural similarity. Segregation of the projected conformers with the cation in some area of the reference sketchmap represents the structural bias introduced by the strong electrostatic interaction with Ca^2+^.

In the case of neutral lysine dipeptide (Fig. 6a), the presence of the Ca^2+^ ion induces relatively small distortions of the stable conformers, that get pushed towards the outer region of the map but are still clearly related to the locally stable structures for the bare molecule. Energies are dramatically changed, with the most stable cluster in the original map being completely absent in the presence of the cation. These observations highlight the importance of sampling high-energy conformers during high-throughput structure searches, since the relative stability can be modulated strongly by external perturbations. In particular, *cis* conformers become energetically more competitive and are topologically closer to the global minima. In the case of protonated lysine dipeptide (Fig. 6b), the same analysis shows an even clearer pattern. All the conformers with Ca^2+^ ions are projected in the lower part of the sketchmap, that correspond to conformers with an extended side chain (see Fig. 4). The Ca^2+^ ion preferably binds to the peptide O atoms, and the electrostatic repulsion with the protonated lysine residue strongly favors extended conformers, contrary to what we observed in the case of the bare molecule. Finally, one sees that for molecular lysine the addition of Ca^2+^ leads to conformers with very different structural motifs from those seen with the bare molecule, which is apparent in the sketchmap projection being concentrated far away from the unperturbed conformers (Fig. 6c). In fact, inspection of the structures shows that Ca^2+^ often triggers the transition to the zwitterionic form, with the cation coupled to the carboxylate group, and the protonated side chain NH$_3^+$ extending as far as possible away from it. In analogy with what was observed for Lennard-Jones clusters [24] and solvated polypeptide segments [79], sketchmap proved to be a powerful tool to analyze the response of the system to external perturbations and changes in the boundary conditions, and—in this specific example—to draw connections between different subsets of a high-throughput molecular database.

### Identifying outliers and checking for consistency

The tools we introduced in this work are useful to address other important issues in data-driven science, such as outlier detection and consistency checks. We have already discussed the importance of detecting groups of outlier structures that are very different from the bulk of the dataset. These unusual items often signal the occurrence of unexpected effects that go beyond the original goal of the database construction effort. In the case of protonated lysine dipeptide, looking for outliers allowed us to reveal the presence of conformers with different chemical connectivity, or of strong H-bonds between the backbone and the charged side chain. Similar observations can also be made in the case of the bare lysine molecule (Fig. 5). Moreover, one can observe a branch at the topmost level of the dendrogram, containing only two conformers. These are the only two cases where a H-bond is formed between the N of the side chain and the H atom of the OH group in the backbone. In the sketchmap, these two conformers are projected on the top, clearly isolated from rest of the groups, and bear the most resemblance to the zwitterionic conformers that are stabilized in the presence of a divalent cation. Obviously, the definition of a group of “outliers” can be more nuanced, and refer to small groups of structures appearing at deeper levels in the hierarchy. Overall, the possibility of clustering together the structures from a large dataset and inspecting a few representative conformers, rather than hundreds or thousands, greatly facilitates the task of identifying trends and spotting interesting or unexpected structures.

**Fig. 7:**
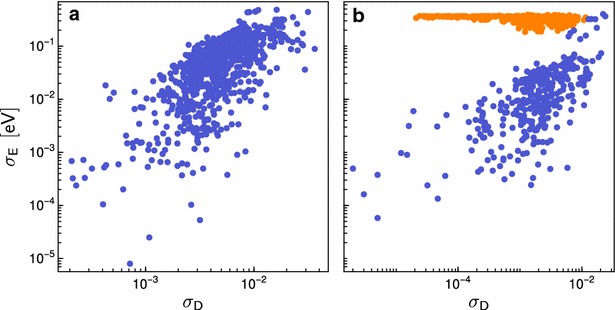
This figure compares the homogeneity of clusters from the protonated lysine dipeptide (**a**) and the bare lysine uncapped (**b**) with respect to properties of their elements. The homogeneity of a cluster is probed using the standard deviation with respect to the distance between each cluster elements, ${{\upsigma }}_D$, and the conformational energy, ${{\upsigma }}_E$. The outliers of uncapped lysine (**b**) were manually highlighted in *orange*

Outliers can signal interesting or important trends, but can also be a red flag for the presence of errors. The importance of database integrity has long been recognized by computer scientists [80–83], and several tools are available to monitor and correct inconsistencies from the technical point of view, in terms of reliability of storing and retrieving data [55–60]. The issue is also crucial when it comes to the maintenance of automatically-generated databases, and to repositories that store data of heterogeneous provenance [1–7]. In these cases, problems have generally little to do with the integrity of the storage, but rather with the consistency of the simulation details of different sets of calculations. Rather, inconsistencies should manifest themselves in the presence of structures that are geometrically very similar, but are associated to very different values of particular properties.

For example the lysine molecule dataset shows signs of this kind of issues, with energies that vary wildly within clusters that are very homogeneous in structure. This problem can be seen from the maps, i.e. when comparing the energy-colored sketchmap in Fig. 5 to the respective maps for the other systems. However, a more robust and easy-to-automate approach to identify structure/property inconsistencies starts from the hierarchical clusters, and compares the structural variability within each cluster $\sigma _D$ (Eq. 8) with the variance of a given property, in this case energy, $\sigma _E$. Looking, for example, at a glassy energy landscape [84], one can observe configurations that are very different from a structural point of view, but have similar energy, giving rise to clusters with large $\sigma _D$ and small $\sigma _E$. The data points in Fig. 7 each represent individual clusters of lysine dipeptide and uncapped lysine, respectively, and illustrate their variation in energy and structure. In the case of lysine dipeptide (Fig. 7a) one sees a clear correlation between the structural and energetical variation of the clusters. The two quantities $\sigma _D$ and $\sigma _E$ are not necessarily strongly correlated, but in general clusters that contain very similar structures also have a low spread in energy. For uncapped lysine (Fig. 7b), however, one can identify a group of points (which we manually highlighted in orange for clarity) that has a distinctively different behavior, with $\sigma _E$ converging to a constant value other than zero as $\sigma _D$ decreases. This kind of feature indicates that the metric based on which structures were classified cannot detect one specific effect that has a dramatic impact on energetics, signaling either a failure of the metric or, as in this case, an inconsistency in the generated data. Further investigation of the lysine molecule dataset revealed that a subset of structures that had been generated at a lower level of theory in the initial stages of the structure-search procedure made their way by mistake into the final dataset. Using this measure of cluster homogeneity on all systems of the amino acid database (see Supporting Information) revealed similar problems also for other molecules, for example Cys, Glu, and Arg. Thanks to this analysis we will be able to identify and rectify mistakes in all the affected datasets and subsequently update the on-line repository [27].

## Conclusion

The increasing use of high-throughput computational screening of materials and molecules, and the compilations of curated databases of the resulting structures and properties, is making more and more urgent to adapt “big data” techniques to the problems that are specific to this field. In this work we have demonstrated how a toolbox of algorithms ranging from hierarchical clustering to non-linear dimensionality reduction can be used to navigate molecular databases, taking as a paradigmatic example some subsets of a database of oligopeptide structures in the gas phase. The software that was used to compute similarity data between molecules, as well as to generate dendrograms and sketch-maps, are open-source and available for download [85, 86].

We find that the use of REMatch-SOAP, a general and unbiased metric to compare different structures based on the combination of pair-wise similarity between molecular environments, makes these techniques particularly insightful. Rather than simply reflecting preconceived notions of what would be the key structural parameters to differentiate molecular conformers, this metric reveals for instance the importance of peptide bond isomerization in describing the high-energy portion of conformational space of oligopeptides, the possibility of changes in chemical connectivity in the course of the ab initio structural search, and the interplay between hydrogen-bonding, backbone dihedrals an electrostatic interactions. Sketchmaps and hierarchical clustering proved to be complementary tools, with representative structures from the main clusters providing an easy way to compare visually different groups of conformers, and the low-dimensional map providing a quick, intuitive tool to verify hypotheses and visualize structure–property correlations.

Assumption-free first-principles molecular-structure search for data generation in combination with dimensionality reduction and clustering for data analysis provide a powerful tool box to study structure formation trends. We could highlight the presence of large portions of configurational space that consist of *cis* isomers of the peptide bond. Albeit energetically unfavorable, these conformers may play an important role in the dynamics of polypeptides. By comparing isolated molecules and their complexes with Ca^2+^, we can also reveal how a strong electrostatic perturbation modifies the energetic landscape of a small molecule—be it by shifting the stability of different conformers, or triggering the formation of new structures that are not observed in the absence of a cation. Furthermore, we also demonstrate the importance of automated analysis techniques in assessing the integrity and the internal consistence of a database, by successfully identifying a subset of structures associated with ill-converged energetics.

All of the techniques we discussed should be readily extendable to heterogeneous databases of molecules and solids, where we expect that the possibility of defining an alchemical kernel within the REMatch-SOAP metric will make it possible to tune the relative weight of composition and structure in determining the notion of similarity. By simplifying the analysis and the interpretation of computational datasets containing thousands or millions of hypothetical compounds, these methods will be crucial to unleash the full potential of computational materials design.

## Additional file

**Additional file 1.** Supplementary file  contains dissimilarity matrix data for all the oligopeptides available in the database including the one discussed in this paper.
